# Baseline Characteristics of Fabry Disease “Amenable” Migalastat Patients in Argentinian Cohort

**DOI:** 10.1155/2024/9293896

**Published:** 2024-02-19

**Authors:** Sebastián Jaurretche, Santiago Alonso, Mónica Calvo, Sebastián Fernandez, Heber Figueredo, Beatriz Galli, Ivanna Marin, Andrés Martinez, Silvia Mattausch, Fernando Perretta, Juan Politei, Juan Ignacio Rolon, Esteban Calabrese

**Affiliations:** ^1^Transplant Department, Sanatorio Parque de Rosario, Rosario, Argentina; ^2^Biophysics and Human Physiology, Instituto Universitario Italiano de Rosario, Rosario, Argentina; ^3^Angel C. Padilla Hospital, San Miguel de Tucumán, Argentina; ^4^STR Cuidadela, Buenos Aires, Argentina; ^5^Instituto de Nefrología y Hemodiálisis, Mendoza, Argentina; ^6^Rheumatology Department, Juan Domingo Perón Hospital, Formosa, Argentina; ^7^San Bernardo Hospital, Salta, Argentina; ^8^Central Hospital, Mendoza, Argentina; ^9^Central Hospital, San Luis, Argentina; ^10^Fresenius Medical Care, Córdoba, Argentina; ^11^Intensive Care Unit, Dr. Enrique Erill Hospital, Belén de Escobar, Buenos Aires, Argentina; ^12^Neurology Department, SPINE Foundation, Buenos Aires, Argentina; ^13^German Hospital, Buenos Aires, Argentina; ^14^INECO, Neurociencias Oroño, Rosario, Argentina

## Abstract

Fabry disease (FD) is a multisystem lysosomal storage disorder induced by genetic variants in the alpha-galactosidase A (*α*GalA) gene. Some FD patients have GLA variants with a reduction in overall *α*GalA enzymatic activity due to mutated proteins with reduced stability, caused by protein misfolding and premature degradation, but the *α*GalA catalytic activity remains conserved (“amenable” genetic variants). To correct this misfolding and to prevent premature degradation, migalastat, a small iminosugar molecule was developed. We report the clinical characteristics of FD “amenable” cohort patients from Argentina, prior to starting treatment with migalastat. Seventeen Fabry adult patients were recruited from 13 Argentinian Centers; 8 males (47.1%) and 9 females (52.9%) were included. All genotypes included were missense-type “amenables” mutations. Some classic FD typical early manifestations were more frequent in patients with “classic” versus “late-onset” FD phenotype (pain, *p*=0.002; cornea verticillata, *p*=0.019). There was a statistically significant difference in estimated glomerular filtration rate in the “classic” versus “late-onset” phenotype (*p*=0.026) but no difference between genders (*p*=0.695). Left ventricular mass was similar between genders (*p*=0.145) and phenotypes (*p*=0.303). Cardiovascular risk factors were present among “late-onset” females (obesity 50% and smoke 25%). In patients who started “de novo” migalastat, the main indications were (i) heart disease, (ii) kidney damage, and (iii) pain, while in “switched from prior enzyme replacement therapy” patients, the most frequent indication was “patient decision;” this coincides with publications by other authors.

## 1. Introduction

Pathogenic genetic variants in the alpha-galactosidase A (*α*GalA) gene (GLA), located on region q22.1 X chromosome, cause the multisystem disorder called Fabry disease (FD) [1]. Reduced or deficient *α*GalA activity is leading to progressive intracellular globotriaosylceramide (Gb3) storage in numerous cells and organs, including the nervous system, kidneys, and heart [1].

“Classic” FD phenotype is presented in men with (i) severely reduced (<1–3% of mean normal) *α*GalA activity; (ii) marked Gb3 accumulation in vascular endothelial cells, cardiomyocytes, smooth muscle cells, and podocytes; and (iii) childhood or adolescent symptoms onset, followed by progressive multiorgan failure with shortening of life expectancy due to premature death [1, 2]. The first clinical manifestations, including neuropathic pain in the extremities (chronic paresthesia and episodic severe pain crises), gastrointestinal (GI) discomfort, hypohidrosis, angiokeratomas, and cornea verticillata typically emerge during childhood or early adulthood [1, 2].

Men with a “Late-onset” FD phenotype present with (i) varying residual *α*GalA activity levels reduced (>3% of the mean normal) and (ii) clinical manifestations usually limited to a single organ, with symptoms onset around the 5^th^ decade of life [1, 2].

In heterozygous FD female patients, disease spectrum severity ranges from asymptomatic to severe “classic” phenotype and is in part dependent on the GLA type variant (genotype) and the X chromosome inactivation (lyonization) profile [1–3].

In Argentina, therapeutic strategies for FD currently include (i) enzyme replacement therapy (ERT): intravenous administration of agalsidase (recombinant *α*GalA; *α* or *β*) and (ii) chaperone therapy which involves the stabilization of the conformation of *α*GalA protein in the endoplasmic reticulum to improve its catalytic ability by oral administration of migalastat, an iminosugar *α*GalA competitive inhibitor. Other therapeutic strategies in development include gene therapy and substrate inhibition therapy.

The real prevalence and incidence of FD are uncertain. While initially reported incidences ranged from 1/476000 to 1/117000 in the general population, newborn screening initiatives have found an unexpectedly high prevalence, as high as 1/3100 newborns in Italy [1]. On the other hand, a high prevalence of FD has been reported in at-risk populations, both in adult men and women on hemodialysis (1/476 and 1/667, respectively), hypertrophic cardiomyopathy (1/476 and 1/667, respectively), and cryptogenetic stroke (1/769 and 1/714, respectively). In Argentina, there are no databases with accurate epidemiological records.

Over 1000 GLA variants have been reported: missense ∼60%, frameshift ∼25%, nonsense ∼8%, and splicing variants ∼7%.

A FD patients subgroup with “missense” type GLA genetic variants can be “amenables” (as explained below) which cause both (i) misfolding and (ii) reduced stability of *α*GalA, so it is prematurely degraded in the trans-Golgi network and cannot reach the cytoplasmic lysosome. Despite this, *α*GalA catalytic activity remains conserved [4, 5]. 1-Deoxygalactonojirimycin (migalastat), a small iminosugar molecule, was developed to prevent *α*GalA premature degradation. Migalastat is capable of binding to the *α*GalA active site, correcting the protein structure misfolding, improving stability, and thus being able to cross the trans-Golgi network without being retained and finally reach the lysosome to fulfill its physiological catalytic function [4, 5]. This pharmacological mechanism, by which a small molecule can help a protein fold correctly, allowing it to enter physiological processing pathways properly was enunciated as “chemical chaperone” [6, 7]. Certain GLA genetic variants specifically cause this *α*GalA structure alteration that can be corrected with migalastat and are called “amenables” (or migalastat responders) variants. In 2016, migalastat was approved by the FDA, and today, it is available as an alternative to ERT to treat FD patients with “amenable” GLA genetic variants [5, 8].

To date, there are no cohorts of “amenable” FD patients reported in Latin America; rather, most studies include these patients from other geographic latitudes. For this reason, in the present work, we report the results of “amenable” FD patients from a Latin American country for the first time.

In the present study, we report the clinical characteristics of FD “amenable” cohort patients from Argentina prior to starting treatment with migalastat. This is the initial report, and the follow-up of this cohort receiving migalastat every 6 months will be reported later.

## 2. Materials and Methods

### 2.1. Patients and Data Collection

We performed a cross-sectional study in 13 FD centers in Argentina (1-Sanatorio Parque de Rosario. 2-Angel C. Padilla Hospital, San Miguel de Tucumán. 3-STR Cuidadela, Buenos Aires. 4-Instituto de Nefrología y Hemodiálisis, Mendoza. 5-Hospital de Alta Complejidad Presidente Juan Domingo Perón, Formosa. 6-San Bernardo Hospital, Salta. 7-Hospital Central de Mendoza. 8-Hospital Central de San Luis. 9-Fresenius Medical Care, Córdoba. 10-Hospital Dr. Enrique Erill. Belén de Escobar, Buenos Aires. 11-Fundación para el Estudio de las Enfermedades Neurometabólicas, Buenos Aires. 12-Hospital Alemán, Buenos Aires. 13-INECO. Neurociencias Grupo Oroño, Rosario) during August 2018 to April 2023. The study was approved by each local committee, and written informed consent was obtained from all patients after oral information.

Inclusion criteria were as follows: FD patients with confirmed diagnosis by the genetic test and “amenable” GLA variants. The study of the genetic variant was carried out by PCR amplification and sequencing of all coding exons and flanking intronic regions from previous DNA extraction from Dried Blood Spot. Reference sequence: NM_000169.2 (ENST00000218516). Exclusion criteria were as follows: (i) FD patients with confirmed diagnosis by genetic test and NO “amenable” GLA variant and (ii) FD patients with inclusion criteria who refused to participate in the study. Quantification of *α*GalA enzymatic activity was performed by the fluorometric method. Decreased or normal enzyme activity was considered at values < than or > than 4.0 nmol/h/l, respectively. Plasma globotriaosylsphingosine (Lyso-Gb3) was quantified by the liquid chromatography method with tandem mass spectrometry.

Plasma and urinary creatinine were determined by electrochemiluminescence (Roche Diagnostics). Urinary albumin was determined by the colorimetric method (Roche Diagnostics). The urinary albumin/creatinine ratio (uACR) was calculated to estimate 24-hour albuminuria [9]. Ratio values 0–30 were considered normal, 30–300 were pathological albuminuria, and >300 were proteinuria in at least two samples. The estimated glomerular filtration rate (eGFR) was calculated using Chronic Kidney Disease Epidemiology (CKD-EPI) [9, 10].

The presence/absence of a neuropathic pain crisis and/or typical acroparesthesias was evaluated by questioning (Brief Pain Inventory–Short Form) and quantitative sensory testing (QST) [1, 2, 11]. The presence/absence of dyshidrosis and typical GI symptoms were evaluated by questioning (Gastrointestinal Symptom Rating Scale) and physical examination [1, 2, 12]. The presence/absence of angiokeratomas was evaluated by a dermatologist specialist in FD [1, 2]. The presence/absence of hearing loss was defined by alterations in the logo-audiometry test [1, 2]. The presence/absence of cornea verticillata was evaluated by ophthalmological examination with a slit lamp, performed by an ophthalmologist with experience in FD [1, 2]. FD cardiac involvement was defined by (i) cardiac arrhythmia typical of FD (electrophysiological disorders in 12-lead electrocardiogram) and/or (ii) left ventricular hypertrophy (LVH) assessed by tissue Doppler echocardiogram and/or cardiac magnetic resonance imaging (MRI); causes of cardiomyopathy other than FD were ruled out [1, 2]. FD central nervous system (CNS) involvement was defined by (i) cerebral white matter lesions in brain MRI angiography and/or (ii) clinical stroke by antecedents during the interrogatory and/or physical examination [1, 2]. Left ventricular mass index (LVMI) was performed by cardiac MRI, and the results expressed in g/m^2^.

### 2.2. Statistical Analyses

Categorical variables were expressed as numbers (percentages) and continuous variables as the mean ± standard deviation (SD). Parametric or nonparametric tests were used according to nominal or continuous variables. The contingency tables were analyzed with the Fisher exact or Chi^2^ test. Differences were considered significant if *p* < 0.05. Statistical analyses were performed with IBM SPSS Statistic Editor 1.8 version.

## 3. Results

Seventeen FD adult patients were recruited from 13 Argentinian Centers; 8 males (47.1%) and 9 females (52.9%) were included (Table 1).

There were no statistically significant differences in the mean age of males (45.8 ± 14.9 years) and females (49.9 ± 14.1 years) (*p*=0.566). The distribution of frequencies and correlations for variables “gender,” “genotype,” “age,” “age at diagnosis,” and “age at symptoms onset” classified by “classic” or “late-onset” FD phenotypes is shown in Table 1.

**Figure 1:**
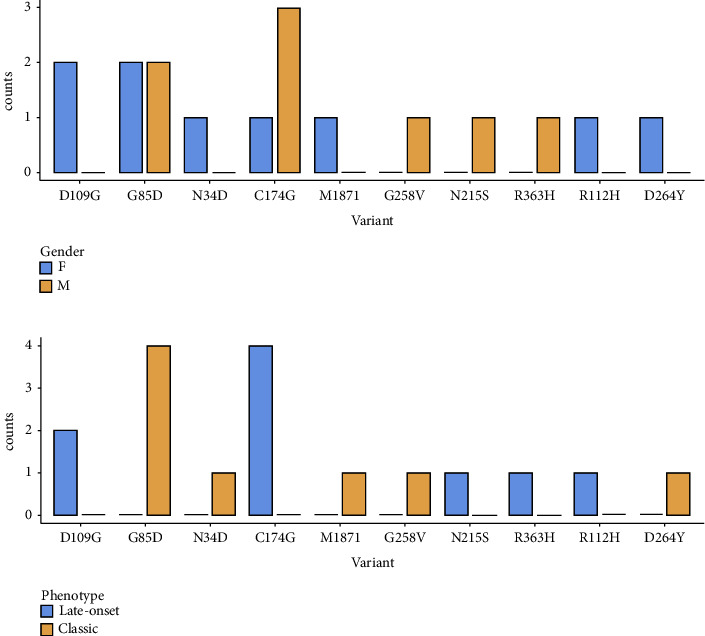
Distribution of genotype frequencies by gender (a) and Fabry phenotype (b). M: males; F: females.

All genotypes included were missense-type “amenables” mutations.  Figure 1 shows the distribution of genotype frequencies by gender (a) and FD phenotype (b).

**Figure 2:**
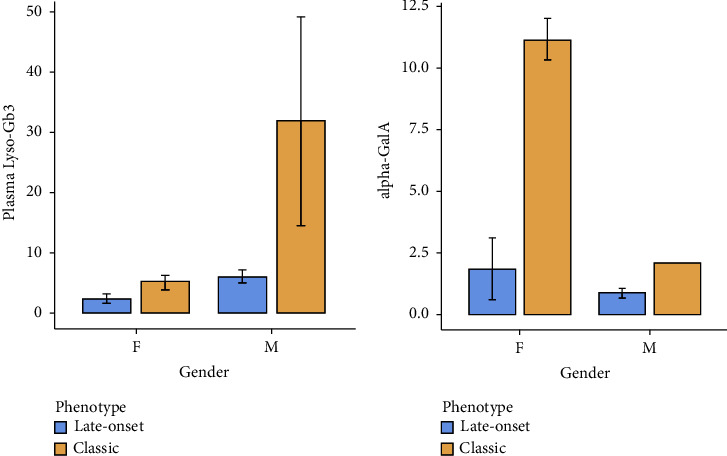
Plasma Lyso-Gb3 levels (a) and *α*-galactosidase-A activity (b) according to gender and Fabry disease phenotype. Alpha-GalA: *α*-galactosidase-A; M: males; F: females.

Plasma Lyso-Gb3 was similar on FD patients “classic” versus “late-onset” phenotype (*p*=0.162) (Figure 2(a)), and *α*GalA enzyme activity was significantly different (*p*=0.006) (Figure 2(b)).

Classic FD typical early manifestations were more frequent in patients with “classical” versus “late-onset” FD phenotype (pain and cornea verticillata with statistical significance, *p*=0.002 and *p*=0.019, respectively; angiokeratomas, diarrhea, and hearing loss without statistical significance, *p*=0.121, *p*=0.121, and *p*=0.611, respectively).

**Figure 3:**
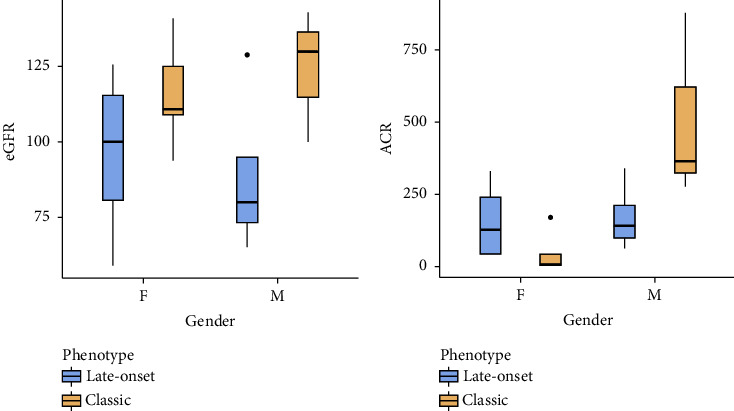
Renal function parameters distribution by gender and Fabry phenotype. eGFR: estimated glomerular filtration rate; ACR: albumin creatinine ratio; M: males; F: females.

The mean eGFR was 96.3 ± 29.4 ml/min/m^2^ among “late-onset” females, 116 ± 18.1 ml/min/m^2^ among “classic” females, 88.5 ± 25.5 ml/min/m^2^ among “late-onset” males, and 124 ± 22.1 ml/min/m^2^ “classic” males (Figure 3(a)). There was a statistically significant difference in eGFR in the “classic” versus “late-onset” phenotype (*p*=0.026) but no difference between genders (*p*=0.695). The mean ACR was 156 ± 140 mg/g among “late-onset” females, 46.1 ± 71 mg/g among “classic” females, 173 ± 106 mg/g among “late-onset” males, and 508 ± 325 mg/g “classic” males (Figure 3(b)). There was a statistically significant difference in ACR in males versus females (*p*=0.049) but no difference between phenotypes (*p*=0.623).

**Figure 4:**
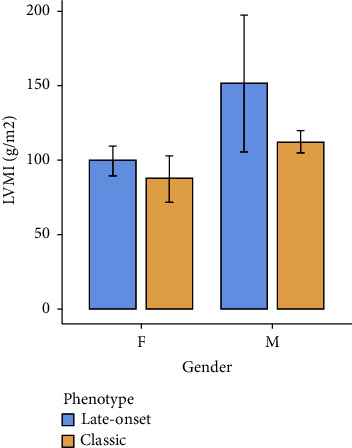
Left ventricular mass in Fabry patients classified by gender and phenotype. M: males; F: females; LVMI: left ventricular mass index.

LVMI was similar between genders (*p*=0.145) and phenotypes (*p*=0.303) (Figure 4) (mean 99.5 ± 19.9 g/m^2^ among “late-onset” females, mean 87.6 ± 35.9 g/m^2^ among “classic” females, mean 152 ± 103 g/m^2^ among “late-onset” males, and 112 ± 13.9 g/m^2^ “classic” males) (Figure 4).

The frequency of cardiac arrhythmia and CNS involvement were similar in patients classified by gender and phenotypes (cardiac arrhythmia between genders *p*=0.522 and between phenotypes *p*=0.375, respectively; CNS involvement between genders *p*=0.929 and between phenotypes *p*=0.929, respectively).

Cardiovascular risk factors were present among “late-onset” females (obesity 50% and smoking 25%).

**Figure 5:**
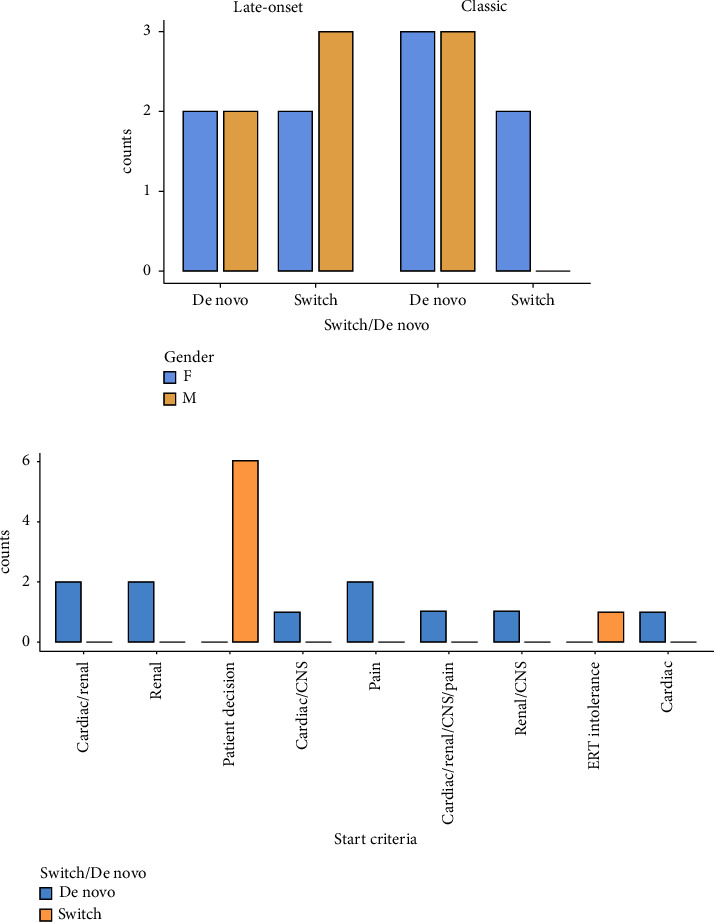
Frequency distribution of variables “switch from ERT to migalastat” and “migalastat de novo” in patients classified by gender and phenotype and start criterion treatment. M: males; F: females.

Figure 5 shows the frequency distribution of the variables “switch from ERT to migalastat” and “migalastat de novo” in patients classified by gender and phenotype (a) and start criterion treatment (b).

## 4. Discussion

Results from a cohort of “classic” and “late-onset” male and female “amenables” FD patients of similar age, diagnosed in adulthood, are presented.

A major methodological limitation of this study is the small number of patients included and its cross-sectional design. The small sample size may affect the distribution of patients in the “classic” versus “late-onset” and “male” versus “female” groups; this could have conditioned the differences in clinical manifestations found in our results. On the other hand, some FD biomarkers can be modified over time; therefore, the cross-sectional design can condition the results; this is the case of Lyso-Gb3.

Lyso-Gb3 has been previously correlated with FD severity and phenotype [13]. Contrary to expectations, in our population, plasma Lyso-Gb3 was similar between both phenotypes (among patients grouped by gender), probably due to an effect of the subjects included distribution, with more patients in the “late-onset” group and within the “classical” population, a greater number of females.

The significant difference in the onset of symptoms is an expected result, since the “classic” phenotype typically presents early symptoms and the “late-onset” not [1, 2].

The significant eGFR difference between “classic” and “late-onset” is an expected result because the classic phenotype typically affects kidney function earlier [1, 2, 14]. Because they present earlier and more severe renal damage, patients with the classic phenotype typically present greater urinary protein excretion [1, 2, 14, 15]. The albuminuria found, similar between both phenotypes, can be explained by the high prevalence of cardiovascular risk factors present in the “late-onset” female group, who theoretically should have less albuminuria due to Fabry nephropathy but have kidney damage from causes other than Fabry [16]. FD cardiac involvement can occur both among adult patients with “classic” phenotypes and in “late-onset” phenotypes with cardiac presentation [1, 2]. Our population did not present differences in the variables “myocardial mass” and “cardiac arrhythmia.”

Central nervous system involvement was similar in patients classified by gender and phenotype. CNS involvement typically occurs in “classic” phenotypes and not in “late-onset” patients, probably the higher cardiovascular risk factors frequency present among “late-onset” females may have modified this result.

In patients who started “de novo” migalastat, the main indications were (i) heart disease, (ii) kidney damage, and (iii) pain, while in the “switched from prior ERT” group, the most frequent indication was “patient decision.” This coincides with publications by other authors [17, 18].

## 5. Conclusions

In Argentina, the indications for starting “de novo” migalastat treatment were heart disease, kidney damage, and pain, while in “switched from prior ERT” patients, the most frequent indication was “patient decision.”

Among Fabry “amenables” patients from Argentina, NOT all major clinical disease manifestations are more frequent among “classic” versus “late-onset” phenotypes.

## Figures and Tables

**Table 1:** Distribution of frequencies and correlations for variables “gender,” “age,” “genotype,” “age at diagnosis,” and “age at symptoms onset” classified by “classic” or “late-onset” FD phenotype.

	Classic FD	Late-onset FD	*p* value
Gender (M/F)	3/5	5/4	0.457
Age	43.11 ± 12.4 ys	52.3 ± 14.9 ys	0.180
Genotype	G85D; N34D; M187I; G258V; D264Y	D109G; C174G; N215S; R363H; R212H	—
Age at diagnosis	36.3 ± 12.8 ys	47.40 ± 14.8 ys	0.116
Age of symptoms onset	19.3 ± 13.4 ys	46.6 ± 13.4 ys	0.003^*∗*^

FD: Fabry disease; M: males; F: females; ys: years; ^*∗*^statistical significance *p* value.

## Data Availability

Data of this work are in the patients' medical records, and the analysis is available in the biostatistics department of the Instituto Universitario Italiano de Rosario, Argentina.

## Abbreviations

*α*GalA: Alpha-galactosidase A
CKD-EPI: Chronic kidney disease epidemiology
CNS: Central nervous system
ERT: Enzyme replacement therapy
FD: Fabry disease
Gb3: Globotriaosylceramide
GFR: Glomerular filtration rate
GI: Gastrointestinal
LVH: Left ventricular hypertrophy
LVMI: Left ventricular mass index
Lyso-Gb3: Globotriaosylsphingosine
MRI: Magnetic resonance imaging
QST: Quantitative sensory testing
SD: Standard deviation
uACR: Urinary albumin creatinine ratio.
